# Exploratory Pressure Injury Prognostic Index for In-Hospital Mortality and Wound-Related Outcomes in Patients Receiving Negative Pressure Wound Therapy: A Retrospective Cohort Study

**DOI:** 10.3390/jcm15187235

**Published:** 2026-09-17

**Authors:** Yüksel Topkaya, Dilara Koç Şeramet, Giray Kolcu, Gökmen Özceylan

**Affiliations:** 1 Department of Orthopaedics and Traumatology, Tekirdag State Hospital, 59000 Tekirdağ, Türkiye; 2 Department of Internal Medicine, Tekirdag Dr. İsmail Fehmi Cumalıoğlu City Hospital, 59000 Tekirdağ, Türkiye; dilarakoc08@gmail.com; 3 Department of Family Medicine, Süleyman Demirel University Faculty of Medicine, 32100 Isparta, Türkiye; giraykolcu@gmail.com; 4 Family Medicine, Department of Palliative Care, Tekirdag Dr. İsmail Fehmi Cumalıoğlu City Hospital, 59000 Tekirdağ, Türkiye; gokmenozceylan01@hotmail.com

**Keywords:** negative pressure wound therapy, pressure injury, prognostic index, retrospective cohort study, palliative care

## Abstract

**Background:** Pressure injuries are common in elderly and immobile patients and are associated with substantial clinical burden and mortality. This study evaluated an exploratory multidimensional prognostic index for in-hospital mortality and wound-related clinical outcomes in patients with advanced pressure injuries receiving negative pressure wound therapy (NPWT). **Methods:** This retrospective, single-center cohort study included 86 patients with stage III–IV pressure injuries treated with NPWT in a palliative care unit. The Pressure Injury Prognostic Index incorporated age, Nutritional Risk Screening 2002 (NRS-2002), Karnofsky Performance Scale (KPS), and Braden Scale scores. The primary outcome was in-hospital mortality, and the secondary outcome was a pragmatically defined favorable wound-related clinical outcome. Mortality discrimination was evaluated using receiver operating characteristic analysis, direct AUC comparisons, and bootstrap-based assessment of internal optimism. Sensitivity analyses examined alternative NRS-2002 weighting values. **Results:** Twenty-seven patients (31.4%) died during hospitalization. Each 10-point increase in the index was associated with higher odds of in-hospital mortality (OR 1.71, 95% CI: 1.23–2.37; *p* = 0.001). The index yielded an AUC of 0.768 (95% CI: 0.659–0.871), with an optimism-corrected bootstrap AUC of 0.765. Its discrimination did not differ significantly from age alone (AUC 0.773; DeLong *p* = 0.890). The post hoc threshold of 192 yielded 77.8% sensitivity and 72.9% specificity. Mortality was higher among patients with scores ≥192 (56.8% vs. 12.2%), whereas favorable wound-related clinical outcomes were less frequent (45.9% vs. 79.6%). **Conclusions:** The Pressure Injury Prognostic Index was associated with in-hospital mortality and wound-related clinical outcomes and may provide a multidimensional summary of clinical vulnerability in patients already receiving NPWT. However, it did not demonstrate superior mortality discrimination compared with age alone. The findings remain exploratory, and external validation, formal calibration assessment, and evaluation of clinical utility are required before routine clinical application.

## 1. Introduction

Pressure injuries are common complications in hospitalized patients. Although they can occur in all age groups, they are especially prevalent in elderly bedridden patients and those with multiple comorbidities. Owing to their high costs and secondary complications, pressure injuries pose a significant burden on healthcare systems. Population aging, increasing healthcare utilization, prolonged care requirements, and pressure injury–related complications contribute substantially to this burden and are associated with complex management, increased healthcare costs, and higher mortality [1].

According to the National Pressure Ulcer Advisory Panel (NPUAP), pressure injuries are defined as localized damage to the skin and/or underlying tissue, usually occurring over bony prominences, as a result of prolonged pressure or pressure combined with shear forces [2]. While the term pressure ulcer traditionally refers to visible tissue loss characterized by an open lesion, pressure injury is a broader concept that encompasses both intact skin damage and deeper tissue involvement that may not yet present as an open wound [2,3,4,5]. The incidence of pressure injuries is affected by many factors, such as patient characteristics and caregivers’ knowledge and experience. It has been reported to be as high as 60% in quadriplegic patients following stroke or spinal cord injury, 65% in patients requiring long-term postoperative care, and up to 70% in patients who remain immobilized for an extended period due to femur fractures [6,7]. Pressure injury management requires a multidimensional approach encompassing prevention, early risk identification, local wound management, nutritional optimization, mobility support, pressure redistribution, and management of systemic comorbidities [8,9]. Importantly, prevention of pressure injury development, assessment of the risk of developing a pressure injury, and prognostic assessment after an advanced pressure injury has already developed represent distinct clinical concepts. Preventive strategies aim to reduce the occurrence of new pressure injuries, whereas risk-assessment instruments identify patients who are susceptible to pressure-related tissue damage. In contrast, prognostic assessment in patients with established stage III–IV pressure injuries focuses on the subsequent clinical course, including mortality and wound-related progression [8]. In clinical practice, pressure injury management includes skin care, topical treatments, reducing factors that delay wound healing (such as smoking and diabetes), pressure redistribution, repositioning and mobilization, nutritional optimization, debridement, protective dressings, electromagnetic energy applications, hyperbaric oxygen therapy, and negative pressure wound therapy (NPWT) [1]. Advanced age is recognized as an important risk factor for pressure injury development [10].

Malnutrition is a significant risk factor that affects the development and healing of pressure injuries. Hospitalized patients are particularly susceptible to malnutrition, and it has been shown that this condition delays wound healing and increases complication rates [11,12,13]. Malnutrition prolongs the inflammatory phase of wound healing, reduces fibroblast proliferation, impairs collagen synthesis, and increases the risk of infection [14]. A recent study on patients receiving palliative care has shown a significant relationship between malnutrition and skeletal muscle loss, which increases clinical frailty [15]. Recent evidence also emphasizes the importance of nutritional assessment and targeted nutritional support in both the prevention and treatment of pressure injuries [16]. Adequate nutritional assessment and support are integral components of multidisciplinary pressure injury care because nutritional vulnerability may adversely affect tissue repair, immune function, susceptibility to complications, and overall physiological reserve.

Therefore, assessing nutritional status is critical for treatment and care planning. The Nutritional Risk Screening 2002 (NRS-2002) is a widely used, valid, and reliable tool for assessing malnutrition risk. Scores range from 0 to 7, with higher scores indicating greater nutritional risk. Advanced age, disease severity, and recent weight loss are important determinants of the risk classification [17]. It has been shown that in patients classified as high risk according to NRS-2002, the risk of developing pressure injuries increases significantly [18]. However, in the present study, NRS-2002 was not used to predict the development of a pressure injury; rather, it was incorporated as a marker of nutritional vulnerability in patients who already had advanced pressure injuries.

Immobility is one of the most important risk factors for the development of pressure injuries. Remaining in a fixed position for a long time leads to pressure-induced ischemia, triggering tissue damage and ulcer formation [3,5]. The Karnofsky Performance Scale (KPS) is a widely used tool for assessing patients’ functional status. The scale is evaluated in increments of 10, ranging from 0 to 100, with 100 indicating full independence. Scores of 0–40 indicate complete dependence, 50–70 indicate partial dependence, and 80–100 indicate full functional independence [19]. As the patient’s mobility, both in and out of bed, increases, the risk of pressure injuries decreases significantly [20]. Within the present prognostic framework, KPS was considered a marker of functional reserve rather than an instrument for predicting pressure injury development.

Various risk assessment tools, such as the Norton, Waterlow, and Braden scales, have been developed to evaluate the risk of pressure injury development [21,22,23,24,25]. The Braden Scale is one of the most commonly used tools for pressure injury risk assessment. This scale evaluates six risk factors: sensory perception, moisture, activity, mobility, nutrition, and friction. Although its ease of use has contributed to its widespread adoption, its sensitivity and specificity values have been reported to vary widely, ranging from 50% to 100%. Although some studies have indicated that it performs satisfactorily, its use alone provides insufficient predictive accuracy, especially in surgical patient groups. Therefore, there is no complete consensus in the literature regarding its predictive validity [26]. Importantly, the primary clinical purpose of the Braden Scale is pressure injury risk assessment rather than prediction of mortality or subsequent clinical outcomes after an advanced pressure injury has already developed. Therefore, its inclusion in the present exploratory index should be interpreted as reflecting baseline multidimensional vulnerability rather than as evidence that the Braden Scale itself is a mortality prognostic instrument.

In Turkey, palliative care units are departments where patients with prolonged stays in intensive care units or other hospital wards, who have developed pressure injuries and are not considered suitable candidates for surgical debridement or definitive surgical wound closure, are managed. This patient population is characterized by multiple comorbidities, substantial functional impairment, and poor overall clinical status. A considerable proportion of these patients are not regarded as suitable candidates for major surgical interventions because of their underlying medical conditions.

In this setting, treatment strategies primarily focus on wound management, symptom control, maintenance of hygiene, and patient comfort, while encouraging the involvement of family members in the care process whenever appropriate. Once an advanced pressure injury has developed, clinical assessment extends beyond prevention and includes evaluation of overall prognosis, realistic goals of care, expected wound-related progression, and the feasibility of continued multidisciplinary wound management [8,16].

The clinical course of patients with advanced pressure injuries is influenced by multiple factors, including age, nutritional status, functional capacity, and pressure injury risk. Therefore, prognostic assessment may assist clinicians in establishing realistic care goals and communicating expected clinical outcomes to patients and their families. In this context, a pragmatic composite assessment incorporating age, nutritional vulnerability, functional status, and pressure injury risk may provide additional information regarding overall clinical vulnerability.

Negative pressure wound therapy (NPWT) is widely used in the management of complex and difficult-to-heal wounds when clinically appropriate [27]. However, the present study does not address the prevention of pressure injuries, indications for NPWT, or patient selection for NPWT. All participants had already been selected for and were receiving NPWT before inclusion in the prognostic cohort. Accordingly, the proposed index was evaluated solely as an exploratory prognostic instrument within this preselected clinical population.

We hypothesized that greater baseline clinical vulnerability, summarized by a composite index incorporating age, nutritional risk, functional status, and pressure injury risk, would be associated with a higher risk of in-hospital mortality and less favorable wound-related clinical progression in patients with advanced pressure injuries receiving NPWT. The primary outcome of the study was in-hospital mortality, and the secondary outcome was a pragmatically defined favorable wound-related clinical outcome during hospitalization.

## 2. Methods

### 2.1. Study Design and Ethical Approval

This study was designed as a retrospective, single-center, observational cohort study evaluating an exploratory, clinically constructed composite prognostic score for clinical outcomes in patients receiving NPWT. The prognostic index was constructed using clinically relevant variables identified from the literature and expert clinical judgment rather than regression-derived predictor coefficients. No variable-selection procedure or multivariable regression-based weighting was applied. Therefore, the present study should be interpreted as an exploratory evaluation of a heuristic composite prognostic score rather than as the development or validation of a formal individual-level prediction model. The index was constructed and initially evaluated within the same cohort. Accordingly, the primary performance estimates represent apparent within-cohort performance; however, bootstrap resampling was additionally performed to assess optimism in the mortality discrimination estimate. This internal assessment does not constitute external validation of the index.

Ethics committee approval for the study was obtained on 25 November 2025 with the number 2025.226.11.23. This study was conducted in accordance with the principles of the Declaration of Helsinki. Patients hospitalized in the palliative care unit of Tekirdag Dr. İsmail Fehmi Cumalıoğlu City Hospital between 1 January 2024 and 30 June 2025 were evaluated retrospectively. Because of the retrospective design of the study and the use of anonymized medical record data, the requirement for informed consent was waived by the Institutional Ethics Committee. All patient data were de-identified prior to analysis, and confidentiality was maintained throughout the study in accordance with institutional policies and the principles of the Declaration of Helsinki.

### 2.2. Participants

The inclusion criteria for the study were the presence of a Stage III or IV pressure injury, prior approval for NPWT by the institution’s multidisciplinary wound care council before study inclusion, complete recording of baseline clinical assessment parameters (age, NRS-2002, KPS, and Braden score) obtained before initiation of NPWT, together with sufficient clinical follow-up data to evaluate the study outcomes (wound-related clinical outcome and in-hospital mortality).

Under the exclusion criteria, patients with malignancy-related (neoplastic) wounds, those with uncontrolled coagulopathy or at risk of active bleeding, the presence of active and uncontrolled osteomyelitis, and patients with incomplete clinical data were excluded from the study.

Patients who developed pressure injuries during hospitalization or were admitted with existing pressure injuries and subsequently received NPWT were included in the study. A total of 86 patients were included in the final analysis (n = 86). The patient was the unit of analysis throughout the study. Pressure injury stage and anatomical location were recorded for the pressure injury undergoing NPWT at the baseline assessment used for study inclusion. All included patients had already been approved for NPWT by the institution’s multidisciplinary wound care council before enrollment in the study. Therefore, the present study was designed exclusively to evaluate prognostic outcomes within a cohort of patients already receiving NPWT and should not be interpreted as addressing patient selection for NPWT, indications for NPWT, or decisions regarding NPWT initiation.

For the purposes of this study, the favorable wound-related clinical outcome was defined as the development of sufficient granulation tissue within the wound bed and progression of the wound to a condition considered suitable for advanced wound management strategies, including secondary closure, grafting, or reconstructive procedures. This endpoint was used solely as a pragmatic retrospective surrogate marker of local wound progression based on the available medical records. It should not be interpreted as evidence that definitive reconstructive surgery was clinically feasible, actually planned, or ultimately performed. Likewise, it should not be interpreted as indicating complete wound closure or overall success of palliative wound care.

### 2.3. Indications and Application of NPWT

The decision to initiate NPWT in our hospital is made by a multidisciplinary council consisting of palliative care, orthopedics, plastic surgery, general surgery, and intensive care specialists based on standard clinical evaluation criteria. The indications for NPWT in patients are assessed by considering parameters such as wound size and depth, presence of infection, tissue perfusion status, and response to previous treatments.

In this context, the main clinical criteria for NPWT application include the presence of a stage III–IV pressure injury, insufficient healing response despite conventional wound care, presence of moderate to high levels of exudate or frequent dressing requirements, a clinical goal of promoting granulation tissue formation, the wound being advanced in terms of size, depth, and tissue loss, and the wound bed being suitable for NPWT application (with controlled necrotic tissue and no active bleeding).

Additionally, control of local infection and the patient’s overall systemic condition not constituting a contraindication for NPWT were also considered during the evaluation process.

Within the applied standard NPWT protocol, polyurethane foam was chosen as the dressing material; the negative pressure level was initially set to −125 mmHg in continuous mode and adjusted to intermittent mode after the second session. Dressing changes were performed every 48–72 h, depending on clinical requirements.

### 2.4. Outcome Assessment and Time Horizon

Outcomes were assessed during the hospital stay. The primary outcome was in-hospital mortality. The secondary outcome was a favorable wound-related clinical outcome, which was assessed at the time of hospital discharge based on routine multidisciplinary clinical evaluation documented in the medical records.

A favorable wound-related clinical outcome was pragmatically defined as the presence of sufficient healthy granulation tissue, adequate wound-bed preparation, and clinical suitability for secondary wound closure, skin grafting, or reconstructive procedures, as determined by the multidisciplinary wound care team.

Because of the retrospective design of the study, no blinded or independent outcome adjudication was performed. Causes of death were extracted retrospectively from the final cause of death documented in the patients’ medical records and were reported descriptively; no independent adjudication of cause of death was performed.

### 2.5. Sample Size

No formal a priori sample size calculation was performed; all eligible patients meeting the study criteria during the predefined study period were included.

### 2.6. Construction of the Pressure Injury Prognostic Index

In this study, an exploratory composite prognostic index based on multidimensional clinical assessment was clinically constructed and evaluated in patients receiving NPWT. The index incorporates key clinical parameters, including age, nutritional status, functional capacity, and pressure injury risk, all of which have been associated with overall clinical status and prognosis in patients with advanced pressure injuries.

The primary purpose of the index was to integrate clinically relevant variables measured on different scales into a single composite score with a consistent direction and comparable magnitude. To achieve this, scaling, directional harmonization, and clinically informed weighting procedures were applied. Accordingly, higher index scores reflect greater overall clinical vulnerability. The index was not designed as a tool for patient selection, initiation of NPWT, or prediction of NPWT-specific treatment response. Rather, it was constructed as an exploratory prognostic instrument intended to reflect the overall physiological reserve and clinical vulnerability of patients already receiving NPWT.

All clinical variables used in the calculation of the prognostic index (age, NRS-2002, KPS, and Braden score) were based on the baseline assessment performed before initiation of NPWT.

Age was included as an untransformed continuous component of the heuristic index to preserve its original clinical scale and maintain simplicity of the composite formulation. No data-driven transformation or nonlinear age term was applied.

We acknowledge that the NRS-2002 includes an age adjustment for patients aged ≥70 years. However, age was retained as an independent component of the composite score because chronological age reflects broader physiological vulnerability, including frailty, immunosenescence, comorbidity burden, and reduced functional reserve, extending beyond nutritional risk alone. Therefore, chronological age and the age-adjusted component of the NRS-2002 were considered related but not identical clinical constructs. Nevertheless, some degree of overlap cannot be excluded, and this should be taken into account when interpreting the exploratory nature of the proposed composite score.

Because the NRS-2002 score has a considerably narrower numerical range (0–7) than the other components of the composite score, a weighting factor of five was applied as a pragmatic heuristic scaling procedure. This adjustment was intended to preserve the clinical contribution of nutritional risk within the composite score and to avoid its influence becoming disproportionately small after combining variables measured on substantially different scales. The weighting factor was not statistically derived or optimized and should therefore not be interpreted as representing an estimated prognostic coefficient. Rather, it reflects a clinically informed heuristic decision based on the recognized importance of malnutrition in the prognosis of patients with advanced pressure injuries.

Because the weighting factor was not statistically optimized, sensitivity analyses were performed using alternative NRS-2002 weighting values to examine the robustness of the index discrimination to this heuristic choice. Future regression-based model-development studies are warranted to determine data-driven component weights and to evaluate the independent contribution of each component.

KPS (ranging from 0 to 100) and the Braden score (ranging from 6 to 23) are variables that are clinically associated with a protective effect. To ensure that all components of the index could be interpreted in the same direction (higher score = worse prognosis), these variables were reversed. For this purpose, (100—KPS) was used for KPS, and (23—Braden), based on the maximum theoretical value of 23, was used for the Braden score. As a result of these transformation and weighting processes, all variables were combined on a common scale, where higher values represented worse clinical status, and a cumulative index was obtained [Table 1].

The index was not derived from statistically estimated coefficients but was clinically constructed based on clinical relevance, scale compatibility, and ease of implementation. It should therefore be regarded as an exploratory heuristic composite score rather than a formal prediction model. Its performance was evaluated within the same cohort in which it was constructed, and the resulting estimates should be interpreted as exploratory and cohort-specific. External validation was not performed.

### 2.7. Statistical Analysis

Statistical analyses were performed using Jamovi software (version 2.3.28; Jamovi Project, Sydney, Australia). Continuous variables were summarized as median and interquartile range (IQR) and categorical variables as frequencies and percentages. Distributional assumptions were assessed using the Shapiro–Wilk test. Comparisons between patients with Pressure Injury Prognostic Index values <192 and ≥192 were performed using the Mann–Whitney U test for continuous variables and Pearson’s chi-square test for categorical variables, as appropriate. Statistical significance was set at *p* < 0.05.

The index was analyzed primarily as a continuous variable, and its association with in-hospital mortality was assessed using univariable logistic regression, with odds ratios (ORs) and 95% confidence intervals (CIs) reported per 10-point increase. Mortality discrimination was evaluated using receiver operating characteristic (ROC) analysis, with area under the curve (AUC) and 95% CI. A post hoc exploratory threshold was identified using the Youden index, and sensitivity and specificity with 95% CIs were calculated.

AUCs for the composite index and its individual components—age, NRS-2002, the KPS-derived component (100 − KPS), and the Braden-derived component (23 − Braden)—were compared using DeLong’s test. Internal optimism in performance was assessed using 3000 bootstrap resamples to estimate optimism-corrected AUC and Brier scores. Because the NRS-2002 ×5 weighting was heuristic, sensitivity analyses were performed using alternative NRS-2002 weights.

For threshold-based analyses, associations of index category (<192 vs. ≥192) with in-hospital mortality and favorable wound-related clinical outcome were expressed as ORs with 95% CIs. Because in-hospital death represented a competing clinical event for the wound-related endpoint, this analysis was considered exploratory.

## 3. Results

**Figure 1:**
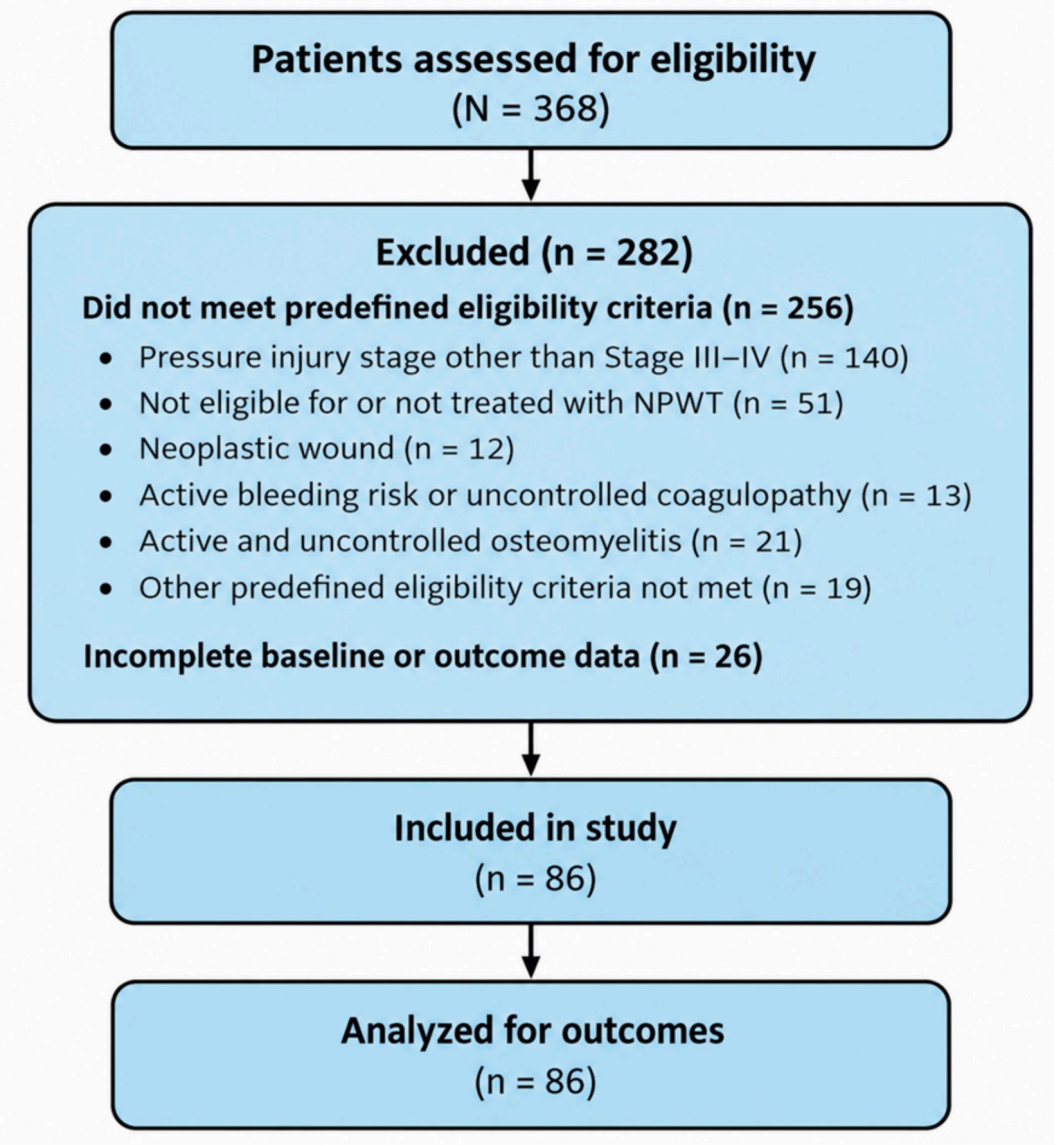
Flow diagram of patient selection and inclusion in the study.

A total of 86 patients who underwent NPWT were included in this study. Of these patients, 39 (45.3%) were male, and 47 (54.7%) were female. The median age was 75 years (IQR: 60–82). The patient selection process is presented in Figure 1. Baseline demographic, clinical, pressure injury, and outcome characteristics of the overall study cohort are summarized in Table 2.

Baseline assessment before initiation of NPWT demonstrated substantial nutritional and functional vulnerability. The median NRS-2002 score was 4 (IQR: 4–5), the median KPS score was 20 (IQR: 10–30), and the median Braden Scale score was 12 (IQR: 10–14) (Table 2). Baseline values according to the exploratory index threshold are presented in Table 3.

During the study, 27 patients (31.4%) died while receiving NPWT. The median age of the deceased patients was 80 years (IQR: 74–89). The most commonly documented cause of death was sepsis (44.4%), followed by pneumonia (29.6%), cardiac causes (18.5%), and multiple organ failure (7.5%).

The most common locations for pressure injuries were the sacral region (46.5%), trochanteric region (27.9%), and heel (15.1%). Stage III pressure injuries were present in 61.6% of patients, whereas stage IV pressure injuries were present in 38.4%.

### 3.1. Discriminatory Performance for In-Hospital Mortality

When analyzed as a continuous variable, the Pressure Injury Prognostic Index was significantly associated with in-hospital mortality. Each 10-point increase in the index was associated with higher odds of in-hospital death (OR 1.71, 95% CI: 1.23–2.37; *p* = 0.001).

In the ROC analysis, the area under the curve (AUC) for discrimination of in-hospital mortality was 0.768 (95% CI: 0.659–0.871). A post hoc Youden-derived exploratory threshold of 192 was identified, with a sensitivity of 77.8% (95% CI: 0.58–0.90) and specificity of 72.9% (95% CI: 0.60–0.83).

**Figure 2:**
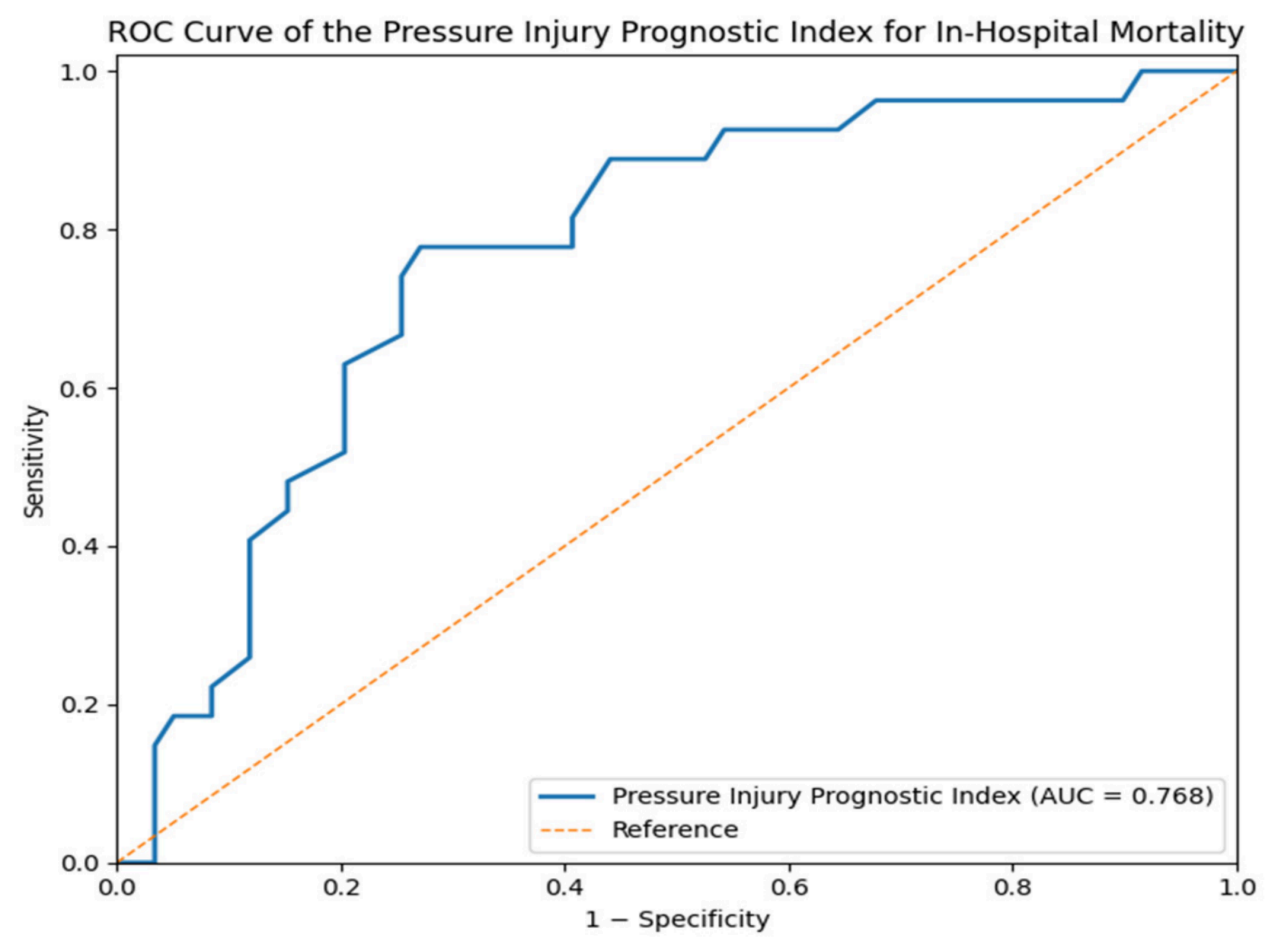
Receiver operating characteristic (ROC) curve of the Pressure Injury Prognostic Index for in-hospital mortality.

ROC analysis demonstrated that the Pressure Injury Prognostic Index showed discrimination for in-hospital mortality within the study cohort (Figure 2). Because the threshold was identified and evaluated within the same cohort, its sensitivity and specificity should be interpreted as internally derived exploratory estimates rather than as externally validated clinical performance.

The discriminatory performance of the composite index was also compared with that of its individual components. The AUC was 0.773 for age, 0.593 for NRS-2002, 0.529 for the KPS-derived component, and 0.600 for the Braden-derived component. DeLong testing showed no significant difference between the AUC of the composite index and age alone (*p* = 0.890). In contrast, the composite index showed significantly greater discrimination than NRS-2002 (*p* = 0.009), the KPS-derived component (*p* = 0.001), and the Braden-derived component (*p* = 0.018) [Table 4].

### 3.2. Bootstrap-Based Internal Performance Assessment and Sensitivity Analysis

Bootstrap-based internal performance assessment using 3000 resamples yielded an optimism-corrected AUC of 0.765, compared with an apparent AUC of 0.768. The apparent Brier score was 0.179, while the optimism-corrected Brier score was 0.188. These findings suggested limited optimism in the apparent discrimination estimate; however, this bootstrap-based assessment does not establish external validity or transportability to other populations.

Because the five-fold weighting of NRS-2002 was based on a heuristic scaling approach, sensitivity analyses were performed using alternative NRS-2002 weights. The original ×5 formulation yielded an AUC of 0.768, whereas alternative lower weighting values produced comparable or modestly higher AUCs. Therefore, the ×5 weighting should be interpreted as a pragmatic scaling choice rather than an empirically optimized prognostic coefficient.

### 3.3. Relationship Between Pressure Injury Prognostic Index, Mortality, and Favorable Wound-Related Clinical Outcomes

Patients were grouped according to the exploratory threshold of 192 (<192 and ≥192). The mortality rate was significantly higher in patients with an index ≥192 than in those with an index <192 (56.8% vs. 12.2%; Pearson’s chi-square test, *p* < 0.001), corresponding to an OR of 9.41 (95% CI: 3.21–27.52).

Favorable wound-related clinical outcomes were observed in 39 of 49 patients (79.6%) with an index <192 and in 17 of 37 patients (45.9%) with an index ≥192. This difference was statistically significant (Pearson’s chi-square test, *p* = 0.001), corresponding to an OR of 0.22 (95% CI: 0.08–0.56) [Table 5].

Because in-hospital death constituted a competing clinical event for the wound-related outcome, this association should be interpreted as an exploratory relationship between greater clinical vulnerability and poorer observed wound-related progression rather than as evidence of an independent effect of the index on wound healing.

## 4. Discussion

In this study, the Pressure Injury Prognostic Index was evaluated as an exploratory multidimensional measure of clinical vulnerability in patients with advanced-stage pressure injuries receiving NPWT. Higher index values were associated with increased in-hospital mortality and a lower frequency of favorable wound-related clinical outcomes. When analyzed continuously, each 10-point increase in the index was associated with higher odds of in-hospital death. The index demonstrated an AUC of 0.768 for in-hospital mortality, while bootstrap-based internal performance assessment yielded an optimism-corrected AUC of 0.765. Importantly, however, the composite index did not demonstrate superior discrimination compared with age alone (AUC 0.768 vs. 0.773; DeLong *p* = 0.890). Therefore, its potential value should not be interpreted as incremental mortality prediction beyond age, but rather as an exploratory multidimensional summary of clinical vulnerability incorporating age, nutritional risk, functional status, and pressure injury-related vulnerability.

This distinction is important because association, discrimination, calibration, and clinical utility represent different aspects of prognostic assessment. The present study demonstrated associations with clinical outcomes and evaluated discrimination, including bootstrap-based assessment of internal optimism; however, formal calibration of absolute outcome probabilities and clinical utility were not assessed. Consequently, the index should not be regarded as a validated individual-level prediction model or clinical decision algorithm, and external validation remains necessary before routine clinical implementation.

The Pressure Injury Prognostic Index includes age, NRS-2002, KPS, and Braden Scale components. The literature consistently shows that the capacity for favorable wound progression and tissue repair decreases with increasing age [28]. In the present cohort, age emerged as a particularly important prognostic factor and showed mortality discrimination comparable to that of the complete index. This finding limits any claim that combining the four components improves mortality discrimination beyond chronological age. Nevertheless, age alone does not directly describe nutritional risk, functional dependence, mobility impairment, or pressure injury-related vulnerability. Thus, the rationale for retaining a composite measure lies in representing several clinically relevant dimensions of vulnerability within a single score rather than in claiming superior discrimination over age alone.

It should also be acknowledged that the NRS-2002 includes an age adjustment for patients aged ≥70 years. Consequently, some conceptual overlap between chronological age and nutritional risk cannot be excluded. In addition, the Braden Scale contains nutrition, activity, and mobility domains that overlap with NRS-2002 and KPS. The components should therefore not be interpreted as independent prognostic constructs, but rather as partially overlapping dimensions of a broader clinical vulnerability phenotype.

Nutritional status was assessed using the NRS-2002 score. In the formula, a fivefold heuristic weighting was assigned to NRS-2002 to preserve the contribution of nutritional risk despite its narrower numerical range. This weighting was based on clinical reasoning rather than statistical coefficient estimation. The present sensitivity analysis further showed that the ×5 weighting was not empirically optimal, as alternative lower weights produced comparable or modestly higher AUCs. Accordingly, the ×5 multiplier should be interpreted only as a pragmatic scaling choice rather than as an estimated prognostic coefficient. Future studies should determine component weighting using appropriately powered multivariable approaches.

The clinical relevance of nutritional and functional vulnerability remains well supported. Tsaousi et al. reported a significant relationship between malnutrition and pressure injury development in hospitalized adults [29], while international recommendations emphasize nutritional assessment and support as integral components of pressure injury prevention and treatment [30,31]. Recent literature similarly supports a multidimensional approach incorporating nutritional support, mobility, pressure redistribution, local wound care, and comprehensive patient assessment [8,16]. Importantly, however, prevention of pressure injury development, assessment of the risk of developing a pressure injury, and prognosis after an advanced pressure injury has already developed are distinct clinical concepts.

The KPS was included in transformed form as (100 − KPS), because lower KPS values indicate greater dependency, poorer functional reserve, and reduced mobility. Boyko et al. highlighted the importance of mobility in pressure injury pathophysiology and healing [20], while O’Toole and Golden described the relationship between poor functional status and reduced rehabilitation potential [19]. Similarly, the Braden Scale was incorporated as a marker of multidimensional baseline vulnerability rather than as a mortality-specific prognostic instrument. Its primary clinical purpose remains assessment of pressure injury development risk. A systematic review and meta-analysis by Huang et al. reported pooled sensitivity and specificity values of 78% and 72% for the Braden Scale, while also highlighting substantial heterogeneity and methodological limitations [26]. Thus, the transformed KPS and Braden components in the present index should be interpreted as directional markers of vulnerability rather than independent causal determinants of mortality or wound progression.

For in-hospital mortality, the composite index demonstrated greater discrimination than NRS-2002, the KPS-derived component, and the Braden-derived component, but not age alone. This direct comparison is central to interpretation of the score. Earlier studies comparing pressure injury risk instruments have also shown variable discriminatory performance. Jun et al. reported AUC values of 0.826, 0.707, and 0.791 for the Cubbin and Jackson, Braden, and Douglas scales, respectively [32], while He et al. reported an AUC of approximately 0.69 for the Braden Scale in surgical populations [33]. However, these instruments were primarily designed for pressure injury risk assessment and are not directly equivalent to the prognostic construct evaluated in the present study.

Bootstrap-based internal performance assessment using 3000 resamples yielded an optimism-corrected AUC of 0.765 compared with an apparent AUC of 0.768, while the apparent and optimism-corrected Brier scores were 0.179 and 0.188, respectively. These findings suggest limited optimism in the observed within-cohort discrimination. Nevertheless, this bootstrap-based assessment does not establish transportability to other clinical populations, and formal calibration and external validation remain required. The post hoc Youden-derived threshold of 192 should likewise be regarded as an exploratory stratification threshold rather than a validated clinical decision boundary.

Previous studies have reported associations between pressure injuries and mortality. Ahtiala et al. identified pressure injury development as an independent predictor of short-term mortality in critically ill patients [34], while Duchesne et al. reported an association between pressure injuries and mortality after adjustment for demographic and comorbidity factors in patients with end-stage renal failure [35]. These studies support the broader prognostic relevance of pressure injuries but do not establish that the present composite index independently predicts mortality or provides incremental prediction beyond age.

A second clinically relevant finding was the association between the index and favorable wound-related clinical outcomes. Favorable outcomes were observed in 79.6% of patients with scores <192 compared with 45.9% of those with scores ≥192. However, in-hospital death represents a competing clinical event because patients who die can no longer subsequently achieve the wound-related endpoint. Therefore, the association between higher index values and poorer wound-related progression may partly reflect the greater mortality burden among more vulnerable patients rather than an independent adverse effect on wound healing.

The wound-related endpoint should also not be interpreted as equivalent to complete wound healing or as evidence of NPWT efficacy. In this study, it represented a pragmatic retrospective marker of local clinical progression, including sufficient granulation tissue, adequate wound-bed preparation, and progression to a condition considered suitable for further wound management. It did not establish that definitive closure, grafting, or reconstructive surgery was subsequently performed.

Poor wound-related clinical progression also poses a significant economic burden on the healthcare system. Gupta et al. emphasized the substantial clinical and financial burden associated with pressure ulcers and the complexity of managing stage III–IV injuries [36].

These observations reinforce the importance of comprehensive multidisciplinary care rather than reliance on a single prognostic score. Nutritional optimization, mobility and repositioning strategies, pressure redistribution, infection management, local wound care, and realistic goal setting remain fundamental components of pressure injury management [8,16]. The proposed index should therefore be considered, at most, an adjunct to multidisciplinary clinical assessment rather than a replacement for individualized evaluation.

NPWT is widely used in the management of chronic, complex, and difficult-to-manage wounds because of its ability to promote granulation tissue formation, reduce wound volume, and control exudate [27]. However, all patients in the present study had already been selected for NPWT by a multidisciplinary wound care team. Therefore, the present findings do not address indications for NPWT, patient selection, treatment initiation or discontinuation, or comparative effectiveness. This preselection also limits generalizability to the broader population of patients with pressure injuries.

In this study, the term “palliative care” describes the treatment setting and does not imply that symptom control was the sole objective for all patients. The palliative care population is heterogeneous, and favorable wound-related clinical outcomes may still be achievable in some patients. At the same time, therapeutic intent for NPWT was not systematically documented at the individual level and may have varied among patients. Accordingly, the wound-related endpoint should be interpreted within this clinical context and should not be generalized across all indications for NPWT. Other important outcomes, including symptom control, exudate and odor management, patient comfort, and quality of life, were not evaluated and should be addressed in future research.

Overall, higher Pressure Injury Prognostic Index values identified patients with greater observed clinical vulnerability, higher in-hospital mortality, and less favorable wound-related progression. However, because the index did not demonstrate superior mortality discrimination compared with age alone, it should not be promoted as an improved mortality prediction model. Its potential value may instead lie in summarizing multiple dimensions of vulnerability within a single pragmatic measure. Prospective multicenter studies should assess calibration, external validity, incremental value beyond age and established comorbidity measures, and whether use of the index improves clinically meaningful decisions or patient-centered outcomes.

## 5. Limitations

This study has several important limitations. First, it was a retrospective, single-center study with a relatively small sample size and a limited number of in-hospital deaths, which may restrict statistical precision and generalizability. Exclusion of patients with incomplete baseline or outcome data may also have introduced complete-case selection bias. Second, the Pressure Injury Prognostic Index was constructed heuristically rather than derived from multivariable regression coefficients, and the fivefold weighting assigned to NRS-2002 was not empirically optimized. In addition, conceptual overlap exists among the index components, particularly between age and NRS-2002 and among the nutritional, mobility, and functional domains represented by NRS-2002, KPS, and the Braden Scale. Third, although bootstrap resampling was used to assess internal optimism in mortality discrimination, external validation was not performed, and formal calibration of absolute risk predictions and clinical utility analyses were not undertaken. Moreover, the index did not demonstrate superior discrimination compared with age alone, and its incremental prognostic value therefore remains unproven.

Fourth, the cohort consisted only of patients who had already been selected to receive NPWT, introducing potential selection bias and limiting applicability to the broader population of patients with pressure injuries. The therapeutic intent of NPWT was not systematically documented at the individual patient level. Detailed individual-level NPWT exposure variables, including total treatment duration, number of sessions, treatment interruptions, and variation in treatment course, were not systematically analyzed. The heterogeneous palliative care setting may also have introduced variability in treatment goals and observation periods. Because wound-related outcomes were assessed at discharge and hospitalization duration varied among patients, the observation period for the secondary outcome was not standardized. Similarly, the in-hospital mortality endpoint was discharge-dependent and did not capture deaths occurring after hospital discharge. Fifth, the wound-related clinical outcome was retrospectively defined as a pragmatic surrogate of favorable wound progression rather than complete wound healing, and in-hospital death represented a competing clinical event that may have influenced the observed association between the index and wound-related outcomes. Stage III and Stage IV pressure injuries were analyzed within the same cohort, and the limited sample size and number of mortality events precluded robust stage-specific prognostic analyses. The source of sepsis could not be reliably classified as pressure injury-related or non-wound-related in all cases; therefore, no causal attribution between pressure injury and sepsis-related mortality was made. Finally, several clinically relevant factors, including detailed comorbidity burden beyond descriptive ACCI reporting, frailty-specific measures, infection-related variables, symptom burden, patient comfort, and quality of life, were not fully incorporated into the prognostic analysis. Prospective multicenter studies with larger samples, external validation, formal calibration assessment, and appropriate competing-risk methods are required before the index can be considered for clinical implementation.

## 6. Conclusions

In this retrospective single-center cohort of patients with advanced pressure injuries already receiving NPWT, the Pressure Injury Prognostic Index was associated with in-hospital mortality and pragmatically defined wound-related clinical outcomes. The index primarily reflects overall clinical vulnerability rather than NPWT-specific treatment response, independent wound-healing capacity, or a tool for patient selection, NPWT initiation, or treatment decision-making. Although the index demonstrated moderate discrimination for in-hospital mortality and showed limited optimism after bootstrap-based internal performance assessment, it did not provide superior discrimination compared with age alone. Therefore, its potential value lies in multidimensional vulnerability stratification rather than incremental mortality prediction. The identified threshold should be interpreted as an internally derived exploratory stratification threshold rather than a validated clinical decision boundary. Accordingly, the findings should be interpreted as exploratory and hypothesis-generating. External validation in larger, prospective, multicenter cohorts, together with formal calibration assessment and evaluation of clinical utility, is required before routine clinical application can be considered.

## Figures and Tables

**Table 1:** Structure and formula of the Pressure Injury Prognostic Index.

**Pressure Injury Prognostic Index = Age + (NRS score × 5) + (100 − KPS score) + (23 − Braden score)**

**Table 2:** Baseline demographic, clinical, and pressure injury characteristics of the overall study cohort.

Characteristic	Overall Cohort (n = 86)
**Age, years, median** (**IQR**)	75 (60–82)
**Sex, n** (**%**)	
Male	39 (45.3%)
Female	47 (54.7%)
**NRS-2002, median** (**IQR**)	4 (4–5)
**KPS, median** (**IQR**)	20 (10–30)
**Braden Scale, median** (**IQR**)	12 (10–14)
**ACCI, n** (**%**)	
0–2	4 (4.6%)
3–4	14 (16.3%)
5–6	25 (29.1%)
7–8	23 (26.7%)
9–10	14 (16.3%)
≥11	6 (7.0%)
**Pressure injury stage, n** (**%**)	
Stage III	53 (61.6%)
Stage IV	33 (38.4%)
**Pressure injury location, n** (**%**)	
Sacral	40 (46.5%)
Trochanteric	24 (27.9%)
Heel	13 (15.1%)
Other locations	9 (10.5%)
**In-hospital mortality, n** (**%**)	27 (31.4%)
**Favorable wound-related clinical outcome, n** (**%**)	56 (65.1%)

**Abbreviations:** ACCI, Age-Adjusted Charlson Comorbidity Index; IQR, interquartile range; KPS, Karnofsky Performance Scale; NRS-2002, Nutritional Risk Screening 2002.

**Table 3:** Baseline clinical characteristics according to the exploratory pressure injury prognostic index threshold.

Variable	Total Population Median (IQR)	Index < 192 (n = 49) Median (IQR)	Index ≥ 192 (n = 37) Median (IQR)
Age (years)	75 (60–82)	63 (52–75)	82 (76–88)
NRS-2002	4 (4–5)	4 (3–5)	5 (4–5)
Karnofsky Performance Scale	20 (10–30)	20 (20–30)	20 (10–20)
Braden Scale	12 (10–14)	13 (11–15)	11 (10–13)

Abbreviations: NRS-2002, Nutritional Risk Screening 2002; KPS, Karnofsky Performance Scale; IQR, interquartile range; NPWT, negative pressure wound therapy.

**Table 4:** Comparison of discriminatory performance of the pressure injury prognostic index and its individual components.

Variable	AUC	95% CI	DeLong *p* vs. Composite Index
Pressure Injury Prognostic Index	0.768	0.659–0.871	—
Age	0.773	0.656–0.878	0.890
NRS-2002	0.593	0.465–0.713	0.009
KPS-derived component	0.529	0.400–0.660	0.001
Braden-derived component	0.600	0.478–0.723	0.018

Abbreviations: AUC, area under the receiver operating characteristic curve; CI, confidence interval; NRS-2002, Nutritional Risk Screening 2002; KPS, Karnofsky Performance Scale. KPS and Braden were directionally transformed as (100 − KPS) and (23 − Braden), respectively, so that higher values indicated greater clinical vulnerability.

**Table 5:** Association of pressure injury prognostic index with in-hospital mortality and favorable wound-related clinical outcomes.

Outcome	Index < 192 (n = 49)	Index ≥ 192 (n = 37)	OR (95% CI)	*p*-Value
In-hospital mortality, n (%)	6 (12.2%)	21 (56.8%)	9.41 (3.21–27.52)	<0.001 *
Favorable wound-related clinical outcome, n (%)	39 (79.6%)	17 (45.9%)	0.22 (0.08–0.56)	0.001 *

* Pearson’s chi-square test. Abbreviations: OR, odds ratio; CI, confidence interval.

## Data Availability

The datasets generated and analyzed during the current study are available from the corresponding author upon reasonable request.

## Abbreviations

ACCI	Age-Adjusted Charlson Comorbidity Index
AUC	Area Under the Curve
CI	Confidence Interval
IQR	Interquartile Range
KPS	Karnofsky Performance Scale
NPWT	Negative Pressure Wound Therapy
NPUAP	National Pressure Ulcer Advisory Panel
NRS-2002	Nutritional Risk Screening 2002
OR	Odds Ratio
ROC	Receiver Operating Characteristic
